# Strengthening the National Sickle Cell Elimination Mission through universal access to point-of-care screening test

**DOI:** 10.1371/journal.pone.0355845

**Published:** 2026-08-13

**Authors:** Beena Joshi, Kavitha Rajsekar, Gaurav Jyani, Kirti Tyagi, Oshima Sachin, R. Revathy, Tejal Varekar, Shankar Prinja

**Affiliations:** 1 Department of Operational and Implementation Research, Regional Resource Hub for Health Technology Assessment, ICMR National Institute for Research on Women’s Health (ICMR-NIRWoH), Mumbai, Maharashtra, India; 2 Department of Health Research, Health Technology Assessment in India, Ministry of Health and Family Welfare, New Delhi, India; 3 Department of Community Medicine and School of Public Health, Postgraduate Institute of Medical Education and Research, Chandigarh, India; World Health Organization, SWITZERLAND

## Abstract

**Objective:**

The study aims to identify the most affordable point-of-care tests and their financial implications for the implementation of the universal screening of sickle cell disease in India.

**Method:**

This study is a rapid health technology assessment from Indian public health system’s perspective using decision tree model for a hypothetical tribal population cohort of 0–40 years in six endemic states. Point-of-care (POC) tests (Hemotype Sc, Sickle Scan, Sickle CERT and Gazelle) were compared with the existing standard of care (SOC) – solubility test with confirmation using high-performance liquid chromatography, for universal screening and early detection of sickle cell disease/traits. The health system cost per case screened and detected were estimated along with budget impact for implementing the screening programs.

**Results:**

The health system cost were higher for all the evaluated POC tests than SOC. The POC screening cost ranged from US$2.38 – US$3.85 per case and US$146.17 – US$230.73 per case detected, compared with US$0.67 and US$42.53 for SOC. However, POC test detected substantially more sickle cell disease cases, incrementally detecting 21,125−41,458 cases. Based on these findings, the National Health Mission directed states to publicly procure POC tests. The price negotiation resulting from the public tendering and competitive bidding resulted in-house manufacturing and validation of these kits lowering their prices substantially which enabled states procuring these POC tests at less than US$ 1.21 per test, potentially yielding US$37.86 million in savings with full coverage.

**Conclusion:**

The rapid HTA provided the state governments with evidence-based guidance to negotiate procurement prices for the POCs which facilitated efficient budget allocation to implement sickle cell screening strategy effectively.

## Introduction

Sickle cell disease (SCD) is a life-threatening genetic hematological disorder. Globally, SCD affects more than 300,000 births every year and is expected to surpass 400,000 by 2050 [1,2]. The most common SCD is sickle cell anemia (SCA), which results from the homozygous inheritance of two sickle β-globin gene variants, one from each parent [3]. The repeated sickling of erythrocytes results in hemolytic anemia, leading to parenchymal injury and chronic organ damage. This causes substantial morbidity and premature mortality [4].

Nearly half of the global burden of SCD is borne by three nations, including India [4,5], where the sickle gene is prevalent among diverse tribal populations [6](5). With over 5200 affected babies annually and 40% prevalence of the sickle cell gene, this condition poses a significant endemic public health challenge in the country [6,7]. According to the study by Hockham et al. 2018, India’s central region has the highest frequency of SCD. The state with the highest number of affected newborns with SCA is Madhya Pradesh, followed by Tamil Nadu, Maharashtra, Gujarat, Odisha and Chhattisgarh [6]. Population migration due to urbanization from endemic areas to other regions, and inter-regional marriages have increased the emergency of the condition, suggesting that in the near future it may not remain endemic to a specific region in India [6].

The Ministry of Health and Family Welfare (MoHFW) of India has identified SCD as one of the ten critical issues among the tribal population that warrants intervention [8,9]. The National Health Mission began addressing hemoglobinopathies in 2016 by releasing comprehensive guidelines for its prevention and management [9]. The Union budget for the financial year 2023−24 announced a mission to eliminate SCD from India by 2047. The National Sickle Cell Anemia Elimination Mission entails a focus on creating awareness, universal screening of 0–40 years endemic populations, and fostering collaboration between the central and state governments [9,10].

As per the national guidelines on hemoglobinopathies, screening for SCD should be performed using a solubility test or point of care, followed by confirmation of positive results using isoelectric focusing or high-performance liquid chromatography (HPLC) [8]. Among newborns, dried blood spot (DBS) followed by high-performance liquid chromatography (HPLC) is performed for screening, and positive cases need to be confirmed after 9–12 months [8]. All these diagnostic tests (except solubility tests) require specialized instruments and trained personnel, thus reducing access due to scarce resources [8,11]. The solubility test exhibits low diagnostic accuracy coupled with an inability to differentiate between trait and disease, emphasizing the need for a viable alternative. The ability to diagnose SCD within minutes using a POC test with high sensitivity and the ability to differentiate between trait and disease will substantially improve timely patient care, enable accurate diagnosis, improve coverage through community screening and decrease the burden on high-throughput equipment [11].

Various POC tests for the diagnosis of SCD are available in India. Hemotype Sc and Sickle Scan are two such popular tests with proven clinical effectiveness [12,13]. Sickle CERT and Gazelle are yet other POC tests, with the added advantage of being developed/manufactured in India [14,15]. Although several studies have investigated the clinical effectiveness of these POC tests, to the best of our knowledge, there has been no evidence in the Indian context evaluating the cost per case of different POC tests available to the standard of care. This rapid health technology assessment was commissioned under the Health Technology Assessment in India (HTAIn) by the Ministry of Health and Family Welfare (MoHFW) to identify the most affordable POC test for SCD. Accordingly, the available POC tests, such as Hemotype Sc, Sickle Scan, Sickle CERT and Gazelle, were assessed in terms of their clinical effectiveness and costs. The financial implications of the introduction of each POC in the public health system are derived through a budget impact analysis.

## Methods

This study was a rapid health technology assessment from Indian public health system’s perspective to evaluate alternative point-of-care (POC) screening strategies for sickle cell disease. The different POC available for screening individuals with sickle cell disease or trait were assessed compared with the current standard of care in the public health system. Evidence on diagnostic performance, epidemiological parameters, resource use and costs incurred by health system were obtained from published literature and publicly available data sources, while primary data collection was undertaken for selected cost components where evidence was unavailable. A decision tree model was developed in Microsoft Excel using these inputs and evaluated over an analytical time horizon of one year to estimate the cost per case detected, cost per case screened and incremental number of cases detected for screening of individuals with sickle cell disease or traits for each point of care (POC) test in comparison with exisiting standard-of-care (SOC) tests. The analysis was undertaken to provide evidence on the comparative costs and expected programmatic outcomes of introducing alternative screening technologies into the Indian public health system and to support evidence-informed decision-making.

### Population

A hypothetical cohort of tribal populations of 0–40 years, from six endemic high disease burden states in India—Madhya Pradesh, Maharashtra, Gujarat, Tamil Nadu, Odisha, and Chhattisgarh—were included [6]. This cohort is as per the guidelines of the National Sickle Cell Anemia Elimination Mission [10]. For the evaluation of the individual POC test screening strategy age group considered varied on the basis of the age-specific proven effectiveness of individual diagnostic tests. Hemotype Sc and Sickle Scan were identified to be effective for newborns [11–13]; thus, newborns were included in the analysis. In the case of Sickle CERT the age considered was from 5 years onwards [14] and for Gazelle the cohort considered was above 6 weeks [15]. The population till 40 years is to be screened for sickle cell disease.

### Intervention and comparator screening test for SCD

The intervention included the 4 Point-of-Care (POC) tests for screening sickle cell disease/traits, namely, Hemotype Sc, Sickle Scan, Sickle CERT and Gazelle, followed by confirmation of screen positives with HPLC. The comparator was the standard of care (SOC), namely, solubility test followed by confirmation of screened positives with HPLC. The National Guidelines on Hemoglobinopathies state screening using solubility or point-of-care tests at the primary, secondary and tertiary levels of healthcare systems. Thus, the decision tree simulated all four screening strategies at the primary, secondary and tertiary levels of healthcare. The confirmatory test using HPLC was simulated at the tertiary level of the healthcare system, as the availability of HPLC is limited to tertiary care facilities alone. The description for sickle cell disease POC and SOC screening tests are presented in the Table 1 below.

**Table 1:** Description for various screening tests for sickle cell disease available in India.

Test	Description	Source
Hemotype Sc	This rapid diagnostic test employs competitive lateral flow immunoassay with monoclonal antibodies to detect Hb A, HbS, and HbC. However, it cannot identify haemoglobin variants like HbD, HbE, and HbF, nor differentiate between HbSS and sickle-β0-thalassemia. Instances of result misinterpretation are reported in cases involving recent blood transfusions. As the kit is unaffected by the high physiological levels of HbF in newborns and selectively target HbA, HbS and HbC; the POC is effective for screening sickle cell disease in newborns.	[11,12]
Sickle Scan	Sickle Scan operates through a sandwich-type lateral flow immunoassay with polyclonal antibodies, identifying HbA, HbS, and HbC. Similar to Hemotype SC, it lacks the capability to detect Hemoglobin variants such as HbD, HbE, and HbF and cannot differentiate between HbSS and sickle-β0-thalassemia. As the kit is unaffected by the high physiological levels of HbF in newborns and selectively target HbA, HbS and HbC; the POC is effective for screening sickle cell disease in newborns.	[13]
Sickle CERT	This rapid, cost-effective, point-of-care method detects both Sickle cell disease and trait. Utilizing a low-power portable system, it relies on the unique light absorption and transmission properties of haemoglobin in a solution. This POC is effective for individuals above 5 years of age.	[14]
Gazelle	The HemeChip cellulose acetate paper-based microchip electrophoresis system includes the Gazelle reader and Cartridge. In addition to detecting HbA, HbS, and HbC like other POCs, it distinguishes HbA, HbF, HbA2, and HbE, enabling differentiation between HbSS and sickle-β0-thalassemia. However, Gazelle, as a facility-based diagnostic method, necessitates trained personnel for administration, processing, and interpretation. Notably, its diagnostic accuracy aligns with the gold standard test (HPLC), making it a viable replacement. The test can be done at 6 weeks onwards and repeated for confirmation of positives with HPLC at 3 months of age.	[15]
Solubility	Solubility test is the current standard of care (SOC) test for SCD. This economical test utilizes phosphate buffer, a hemolysing agent, and sodium dithionate, causing turbidity due to HbS crystallization. However, its low sensitivity, specificity, and in-applicability in newborns render it an inefficient screening method, prompting the need for an alternative.	[8,16]
HPLC	This method uses elution of hemoglobin, bound to a solid phase. It is a very precise, reproducible test, therefore is the gold standard test for various hemoglobinopathies including SCD. In the current SOC it used alongside solubility for confirmatory test. However, it requires very complex and expensive infrastructure and trained manpower.	[8,16]

### Cost data

The costs were obtained using a mixed methodology approach, and both primary and secondary data sources were used. The cost of screening estimated for the POC test and SOC test strategy included both the cost of the test kit/device and the associated health system costs incurred at different levels of healthcare delivery, the primary, secondary and tertiary levels, using previously published literature and costing database [17,18]. The capital and shared resource costs comprised infrastructure, equipment, utilities, maintenance, consumables, administrative overheads and personnel time associated with conducting screening services. These costs were derived from standard treatment guidelines, Costing of Health System in India (CHSI) study and the National Health System Costing Database for India. The cost of the POC and SOC tests kit/devices was obtained from the manufacturer or public procurement system. The cost of confirming positive results by HPLC at the tertiary level was estimated through a primary costing approach. The cost data derived from generalised health system costing databases and published literature were applied to six high burden tribal endemic states from which the study cohort was estimated which is targeted under the National Sickle Cell Anemia Elimination Mission. The higher per-person capital and shared resource costs observed at secondary-level facilities reflect comparatively lower service volumes and reduced economies of scale relative to tertiary facilities. The data for the cost parameters used in this study were accessed for research purposes between 08/12/2023 and 10/06/2024.

The cost of conducting HPLC tests at tertiary hospitals was estimated using a primary costing study conducted at a tertiary hospital in the state of Maharashtra using the Indian costing tool modified for the current study. The cost data, specifically for various components such as machines, equipment, kits, reagents, consumables, sample transport, maintenance, etc., were collected from hospital records, annual reports and staff interviews for the financial year 01/04/2022–31/03/2023. The quantities of these input and service outputs were measured. The cost data was annualised. The the outpatient unit at tertiary level facility which included the capital and share cost including the infrastructure, equipment, utilities, maintenance, consumables, administrative overheads and personnel time associated with conducting test was obtained from the costing database. The unit cost for conducting HPLC tests at tertiary facilities was estimated on the basis of the cost specific to the HPLC test obtained through primary costing and the cost of human resources, capital and overhead at tertiary facilities from secondary sources. This data was accessed for research purpose on 08/08/2023. All costs were analysed in Indian rupees (INR) at 2023 prices and converted to USD for presentation (1 US$ = INR 82.57) [19]. The cost parameters considered for the analysis are listed in Table 2 below.

**Table 2:** Input parameters specific to cost.

Input parameters	Value (in US$)	Source
**Hemotype Sc and Sickle Scan**		
Cost of capital and shared resources per person at primary level	0.46	[17]
Cost of capital and shared resources per person at secondary level	1.10	[18]
Cost of capital and shared resources per person at tertiary level	0.71	[18]
Cost per test of POC for Hemotype Sc and Sickle Scan	2.57	Obtained from Manufacturer
**Sickle CERT**		
Cost of capital and shared resources per person at primary level	0.46	[17]
Cost of capital and shared resources per person at secondary level	1.10	[18]
Cost of capital and shared resources per person at tertiary level	0.71	[18]
Cost per test of POC for Sickle CERT	1.82	[20]
**Gazelle**		
Cost of capital and shared resources per person at primary level	0.46	[17]
Cost of capital and shared resources per person at secondary level	1.10	[18]
Cost of capital and shared resources per person at tertiary level	0.71	[18]
Cost per test of POC for Gazelle kit	2.18	Obtained from Manufacturer
Cost of Gazelle machine	3027.59	Obtained from Manufacturer
**Solubility test**		
Cost of capital and shared resources per person at primary level	0.34	[17]
Cost of capital and shared resources per person at secondary level	0.64	[18]
Cost of capital and shared resources per person at tertiary level	0.41	[18]
Cost per test of POC for Solubility test	0.21	MoHFW Procurement price
**HPLC test**		
Cost of capital and shared resources per person at tertiary level	0.71	[18]
Cost of HPLC test per person	4.04	Primary costing

### Effect data

The number of cases screened and detected was estimated using each strategy. The total number of eligible populations for each of the scenarios were obtained from the 2011 Indian Census data for 6 high prevalent states [21]. The estimation of individuals with sickle cell disease within this population was based on the disease prevalence. The proportions of individuals at each level of healthcare were determined on the basis of the utilization of services. Studies assessing the proportion of individuals availing healthcare services at primary level have revealed that 70% of services are utilized at primary care levels (Rural Health Centres) in India [22,23]. At the primary level, SCD screening is both facility-based and community-based active screening, so most of the population (70%) will be screened at this level. The screening at the secondary and tertiary levels is primarily opportunistic and referral-based. The populations at the tertiary and secondary levels were determined on the basis of the utilization and availability of HPLC, which were 20% and 10%, respectively. The sensitivity and specificity of the tests were used to calculate true positives, true negatives, false positives and false negatives, which in turn provided the total number of detected and undetected cases. The data for the input parameters used in this study were accessed for research purposes between 08/12/2023 and 10/06/2024. The input parameters are listed in Table 3 below.

**Table 3:** Input parameter specific to effects.

Input parameters	Value	Source
Population cohort Hemotype SC	4.11 crores	[21]
Population cohort Sickle Scan	4.11 crores	[21]
Population cohort Sickle CERT	3.51 crores	[21]
Population cohort Gazelle	3.99 crores	[21]
Prevalence of sickle cell diseases (per 100)	1.67%	[6]
Prevalence of sickle cell trait (per 100)	16%	[6]
Sensitivity of solubility test	93.8%	[24]
Specificity of solubility test	99.9%	[24]
Sensitivity of gold standard test (HPLC)	96.8%	[16]
Specificity of gold standard test (HPLC)	100.0%	[16]
Sensitivity of Hemotype Sc	97.3%	[11]
Specificity of Hemotype Sc	99.9%	[11]
Sensitivity of Sickle Scan	97.8%	[25]
Specificity of Sickle Scan	99.2%	[25]
Sensitivity of Sickle CERT	97.4%	Estimated
Specificity of Sickle CERT	97.5%	Estimated
Sensitivity of Gazelle	100.0%	[15,25]
Specificity of Gazelle	99.0%	[15,25]
Proportion of tests conducted at Primary Level	70%	[22,23]
Proportion of tests conducted at Secondary Level	20%	[22,23]
Proportion of tests conducted at Tertiary Level	10%	[22,23]

### Data analysis

**Fig 1:**
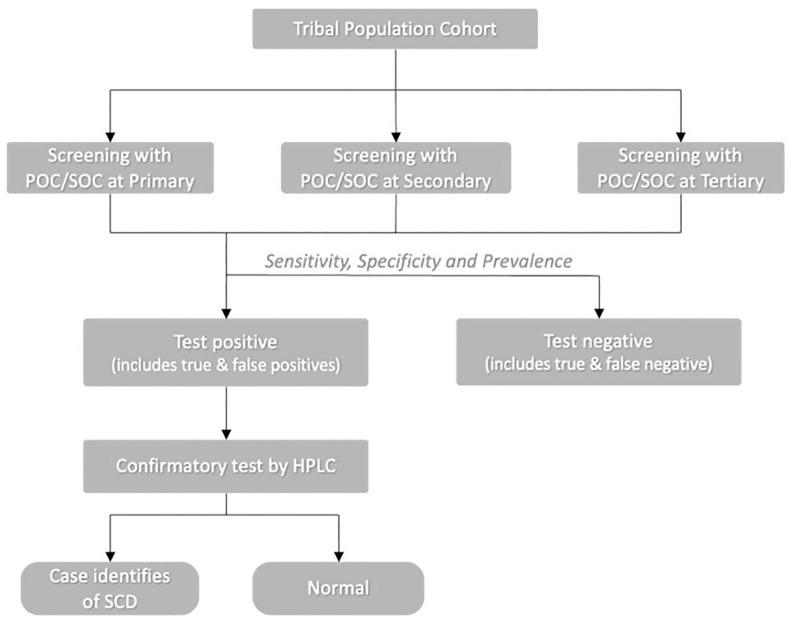
Decision tree for screening of sickle cell disease/trait patients using point of care (POC) and standard of care (SOC).

On the basis of the levels of service utilization at various levels of healthcare, the eligible tribal population that could be screened at primary, secondary and tertiary levels of healthcare was determined, and accordingly, the costs of screening at each level were computed. The strategy involves screening using the POC test followed by confirmation with HPLC. Using the prevalence of sickle cell disease and traits and the sensitivity and specificity of the POC test, the number of individuals who could be screened as positive (true and false positive) was computed. For confirmation of screened positives by HPLC, the sensitivity and specificity of HPLC were used to compute the number of individuals with disease and traits (true positives) among all the positive cases. The decision tree model for screening sickle cell disease/trait is described in Fig 1. The cost of screening at tertiary levels and the unit costs of HPLC were utilized to estimate the costs for the confirmation test. The health system costs were estimated from the summation of the costs of screening at primary, secondary and tertiary along with the costs for confirmatory tests by HPLC. The incremental cost was estimated on the basis of the additional healthcare resources for the POC test compared with the SOC test for screening the eligible population cohort. The unit costs per test screened were determined using the total health system costs of screening and the number of individuals screened. The total number of individuals estimated for disease and trait through the model was used to compute the unit cost per case detected.

### Sensitivity analysis

The probabilistic sensitivity analysis (PSA) was conducted to evaluate the robustness of the data. Using the Monte Carlo simulation method, 1000 simulations were run for various parameters. The lower and upper limits of 95% CI intervals were ascertained corresponding to 2.6 percentile and 97.5 percentile values.

### Budget impact analysis

The budget impact of implementing sickle cell disease/trait screening strategies for POC tests in public health facilities was assessed for six endemic states such as Madhya Pradesh, Maharashtra, Gujarat, Tamil Nadu, Odisha, and Chhattisgarh with a major burden of sickle cell disease in India. The projection was estimated based on population coverage of 50%, 70% and 100%. The implementation cost included the cost of POC test, consumables, training and the IEC as it would be undertaken with existing manpower and infrastructure. The mean cost of all the 4 POC tests was considered. The budget was estimated for two different scenarios; one with the mean cost for POC test which is based on the market price and the other negotiated price at which states are procuring. The unit cost of implementation of the screening strategy at public health facilities along with the estimated population coverage was used to determine the additional budgetary requirements for implementation at the public health facilities. The budget impact in terms of cost savings of implementing the POC diagnostic test at market price and negotiated price has been estimated.

## Results

### Cases detected and incremental cases detected

The POC test for sickle cell diseases/traits detects more cases than does the SOC test of solubility and HPLC. However, this includes both true and false positives, and the number of false positives was also high for the POC tests. The POC test is also able to differentiate and inform individuals with disease and traits, which is not the case with the SOC, where both are detected without differentiation. For the study population cohort, the number of cases detected, including both diseases and traits, by the POC test gazelle was 0.668 million, the number of sickle SCAN cases was 0.671 million, the number of hemotype Sc cases was 0.668 million, and the number of sickle CERT cases was 0.571 million. The observed differences in estimated diagnostic outcomes across screening tests are driven by small variations in test sensitivity and specificity, which when applied to a large screened population cohort, result in substantial differences in the absolute number of detected, undetected and incorrectly classified cases (false positives and false negative).

Compared with SOC, the POC tests incrementally detected more cases of sickle cell disease or traits with Gazelle (41,458 cases), Sickle Scan (27,467 cases), Hemotype Sc (24,034 cases) and Sickle CERT (21,125 cases), which were otherwise undetected using SOC. The incremental number of true positive cases of sickle cell disease detected by the Gazelle is 6% greater than that detected by the SOC and Sickle CERT is 4% greater than that detected by SOC. In the case of Hemotype Sc and Sickle SCAN, which also detect sickle cell disease in newborns, 4% more true positive cases, are detected compared with the solubility test. The number of cases detected and undetected with each intervention test (Hemotype Sc, Sickle Scan, Sickle CERT, Gazelle) compared to the Solubility test, both followed by confirmation of positives with HPLC for screening program is provided in the Table 4 below.

**Table 4:** Measures of diagnostic accuracy and number of cases detected and undetected with each intervention test (Hemotype Sc, Sickle Scan, Sickle CERT, Gazelle) compared to the Solubility test, both followed by confirmation of positives with HPLC for screening program.

Parameters	Hemotype Sc Vs Solubility test		Sickle Scan Vs Solubility test		Sickle CERT Vs Solubility test		Gazelle Vs Solubility test	
	Hemotype Sc*	Solubility test *	Sickle Scan*	Solubility*	Sickle CERT*	Solubility test*	Gazelle*	Solubility test *
**Cohort Size**	41118211		41118211		35138004		39954483	
**True positives**	668134	644100	671567	644100	571547	550422	668670	627212
**False positives**	40432	4043	323452	4043	863779	3455	393714	3937
**True negatives**	40391105	40427493	40108085	40427493	33687419	34547744	38977763	39367541
**False negatives**	18540	42573	15107	42573	15256	36381	0	41457
**Number of cases detected**	668134	644100	671567	644100	571547	550422	668670	627212
**Number of cases undetected**	18540	42573	15107	42573	15256	36381	0	41457
**Incremental number of cases detected by the intervention**	24034		27467		21125		41458	

*Positives were confirmed with HPLC.

### Health system cost and incremental cost

The health system cost of using a POC test for screening the entire cohort of tribal populations for sickle cell disease for each strategy is estimated to be US$ 154.28 million for Gazelle, US$ 134.02 million for Sickle SCAN, US$ 132.65 million for Hemotype Sc and US$ 90.26 million for Sickle CERT. The health system cost for SOC varied with each POC test on the basis of the different cohort sizes based on the different age groups of each strategy; for example, for the population cohort aged 0–40 years (Hemotype Sc and Sickle SCAN), the cost was estimated to be US$ 27.57 million; for the cohort aged 5–40 years (Sickle CERT), the cost was US$ 23.56 million; and for the cohort aged 6–40 years (Gazelle), the cost was US$ 26.84. The health system cost for screening using the POC was higher than that for the SOC. The incremental health system cost for each POC test for sickle cell disease screening compared with the standard of care for Gazelle was US$ 127.44 million, that of Sickle SCAN was US$ 106.45 million, that of Hemotype Sc was US$ 105.09 million, and that of Sickle CERT was 66.7 million. The health system cost of screening for each screening strategy is presented in Table 5.

**Table 5:** Cost measures with each intervention test (Hemotype Sc, Sickle Scan, Sickle CERT, and Gazelle) compared to the Solubility test, both followed by confirmation of positives with HPLC for screening program.

Parameters	Hemotype Sc Vs Solubility test		Sickle Scan Vs Solubility test		Sickle CERT Vs Solubility test		Gazelle Vs Solubility test	
	Hemotype Sc	Solubility test	Sickle Scan	Solubility test	Sickle CERT	Solubility test	Gazelle	Solubility test
**Health system and Incremental cost for the screening program (in millions USD)**								
**Health system cost for screening SCD**	132.65	27.57	134.02	27.57	90.26	23.56	154.28	26.84
**Incremental cost for screening SCD**	105.09		106.45		66.70		127.44	
**Cost per cases screened and detected (in USD)**								
**Cost/ case screened**	3.23 (2.41, 4.07)	0.67 (0.50, 0.84)	3.26 (2.44, 4.10)	0.67 (0.5, 0.84)	2.57 (1.69, 4.03)	0.67 (0.5, 0.84)	3.85 (2.88, 4.78)	0.67 (0.50, 0.84)
**Cost/ case detected**	198.55 (150.09, 246.12)	42.53 (32.13, 53.42)	199.57 (149.05, 248.24)	42.53 (32.13, 53.42)	157.92 (108.34, 191.72)	42.53 (32.13, 53.42)	230.73 (170.16, 290.50)	42.53 (32.13, 53.42)

### Cost per case screened and detected

The cost per case screened with Gazelle was US$ 3.85, Sickle Scan was US$ 3.26, Hemotype SC was US$ 3.23, and Sickle CERT was US$ 2.57. The cost per case screened for all POC tests is greater than the cost per case screened for the SOC, which is US$ 0.67. The cost per case detected for Gazelle was US$ 230.73, Sickle Scan was US$ 199.57, Hemotype SC was US$ 198.55, and Sickle CERT was US$ 157.92. Compared to all the POC tests, the cost per case detected for SOC is lower at US$ 42.53. Although the cost per case detected was higher for the POC tests, the number of cases detected was greater for the POC tests than for the SOC test. The cost per person screened and the cost per case detected are estimated and presented in Table 5.

### Translation to policy

This rapid health technology assessment commissioned under the Health Technology Assessment India was critical to supplement the efforts of the Ministry of Health and Family Welfare (MoHFW) under the National Sickle Cell Anemia Elimination Mission for universal screening and early detection of SCD by making affordable POC tests available at every level of health service delivery. The results of the assessments were approved by the Technical Appraisal Board of the Health Technology Assessment in India (HTAIn). The policy briefs were shared with the Ministry of Health and Family Welfare, India (MoHFW), who provided recommendations on the use of Hemotype Sc, Sickle Scan, and Sickle CERT at the community level screening and Gazelle at the facility level screening, followed by confirmation of positive results with HPLC. Following the HTA recommendation, the National Health Mission instructed state health departments to procure the diagnostic kits through public tendering. This encouraged inhouse manufacturing and validation of similar rapid POC test kits. Its enabled competitive bidding and lowering the prices of the POC test kits which resulted in states procuring POC tests less than US$ 1.21 per test. Many states have ensured efficient budget allocation and started procurement and use of these POC tests for sickle cell screening in their respective states [26].

### Budget impact analysis

The population cohort of the tribal population in the six endemic states for the age group 0–40 years was 41.1 million. The estimated budget provided the additional resource spending on the screening utilizing the current infrastructure and human resource. The budget impact for the POC test screening with the POC diagnostic test at the market price and negotiated price is provided in the Table 6 below. The price negotiation resulting from the competitive procurement process will provide a total cost saving of US$ 37.86 million with 100% coverage for screening of sickle cell disease/trait.

**Table 6:** Budget Impact for Sickle POC test.

SCD Screening budget with POC test	Varied population coverage *(In US $ millions)*		
	50%	70%	100%
**POC tests market price**	64.86	88.14	123.05
**POC tests at negotiated price (less than $1.21)**	44.90	61.02	85.19
**Cost savings**	19.96	27.12	37.86

## Discussion

The availability of timely screening and detection of sickle cell disease has considerable implications for the control and treatment of sickle cell disease morbidity and mortality. Specifically, in the case of newborns, a significant decrease in patient mortality is indicated when newborn screening is coupled with extensive follow-up and education [27]. In countries such as India, where SCD is highly prevalent and specialized laboratory diagnostics are predominantly available only at private or large referral hospitals, the diagnosis of patients is a major challenge [28].

The HPLC test, despite being the gold standard, is not available at lower levels of healthcare facilities due to the expensive machinery and expertise required for it, limiting its access to the marginalized population and reliance on screening with a less accurate test [28]. At lower-level primary healthcare facilities, the most widely used test is the solubility test; however, this test has low clinical utility, as it does not distinguish the sickle cell trait from sickle cell disease [28]. Furthermore, all positive cases identified through solubility tests are referred, or their samples are sent on filter paper for confirmation through HPLC. By using POC, the differentiation of disease or trait can be obtained at the primary level, resulting in early detection and onset of treatment for diseased cases and counselling of traits. This early detection and treatment is critical for improving health outcomes among sickle cell individuals. The ability of POC tests to differentiate sickle cell disease from trait at the initial screening stage may additionally reduce unnecessary referrals and lower the burden on tertiary facilities for confirmatory testing compared with the solubility test strategy. The POC tests are easy to handle and highly sensitive. Hemotype Sc, Sickle Scan and Sickle CERT are strip-based tests with no major requirements in terms of machinery, and trained personnel could be indicated for door-to-door community-based screening by ASHAs, which is an all-inclusive universal method of screening that would be most appropriate for tribal populations in India [11–14]. The other test, the Gazelle test, requires a machine and kit; hence, for this method, a facility-based diagnostic strategy has been suggested. Although Gazelle demonstrated comparatively higher screening costs compared to other POC tests, the availability of Gazelle at lower level of facilities has a major advantage, as their performance is similar to that of HPLC. Compared with HPLC, the Gazelle is a cheaper machine that requires no expert training for manpower and requires less processing time. It can additionally enabled identification of other haemoglobin variants such as HbE and HbA2. [15]. Therefore, public health facility screening of high-risk populations with Gazelle would improve access to screening using a highly sensitive test where access to HPLC is limited.

When comparing the cost per case of POC test with that of the SOC test, the cost per case screened and cost per case detected was greater for all four available POC tests. Thus, procurement of these POC test at market prices would require higher additional budgetary resources. However, the better diagnostic accuracy of these intervention tests, which informs on-spot whether a trait or disease, makes it a more reliable screening method. This is critical to detect and manage cases of SCD in endemic populations. Utilising the rational of higher cost per case provided by this rapid health technology assessment the states were able to negotiate prices to procure additional POC test which resulted in significant cost saving compared to the market price. This is an essential step to strengthen the universal screening under the National Sickle Cell Anemia Elimination Mission. The rapid HTA provided the state governments with evidence-based guidance to negotiate procurement prices for the POCs which facilitated efficient budget allocation to implement sickle cell screening strategy effectively. The availability of POC test screening strategies is critical to increasing the number of screening opportunities and enabling active screening at every level of health service delivery, including the community level. Sickle cell anemia is a significant public health challenge, particularly in states where the burden of disease is high. Hence, early diagnosis is crucial to provide appropriate management for reducing the burden of disease and death due to SCD.

### Strengths

The present study is the only rapid health technology assessment to comprehensively analyze the cost and effects of the available POC test and SOC for screening SCD in India. Most of the previous studies have only assessed the clinical effectiveness of various screening tests for SCD. The health technology assessment provides a comparison of the available POC with SOC. It provides evidence for decision makers to adopt the POC screening strategy. The study also analysed and presented the budget impact assessment of implementing the screening for SCD. The literature references have been used from the India study settings to ascertain the input parameters used for analysis. Expert opinions and suggestions were obtained and this assures the quality of the analysis.

### Limitations

The current analysis measures the effects in terms of cases detected by the test. There are many more benefits of identifying sickle cell disease and traits, which POC tests impart. These benefits will be reflected in terms of better health outcomes and lower management costs for complications because of early detection and prompt treatment in SCD patients, as well as in the forthcoming birth cohort of SCT patients. In the case of Gazelle, the additional benefit of detecting thalassemia along with SCD was not considered in the present study due to the unavailability of literature evidence to carry out the analysis. Therefore, the analysis would have underestimated the health benefits of these tests. Additionally, a large proportion of the population would know they are disease free through community-based screening, the benefits of which have not been accounted in this study. The additional health system burden and cost associated with more cases of SCD patients have not been estimated for this analysis.

## Conclusions

The ability to diagnose SCD at the point of care (POC) within minutes using a low-cost, portable device will substantially improve patient care by enabling more accurate differential diagnosis in patients. This will tremendously assist in more accessible and affordable community-based screening. The rapid HTA provided the states with the evidence-based procurement and implementation making sickle cell screening affordable and available at every level of health service delivery. The POC tests provides an opportunity for SCD screening with higher diagnostic accuracy and more cases detected, which otherwise would have remained undetected using the SOC. Importantly, in addition to providing accurate, quick results, the easy use of POC tests enables improved access to tests in community settings, which could in turn improve outcomes. The utilization of these POCs is critical to meet the objectives of the National Sickle Cell Anemia Elimination Mission for universal screening of approximately seven crores tribal people aged 0–40 years and to eliminate sickle cell anemia by 2047.

## Abbreviations

POC: Point of care
HPLC: High performance liquid chromatography
SOC: Standard of care
SCD: Sickle cell disease
SCA: Sickle cell anemia
MoHFW: Ministry of health and family welfare
DBS: Dried blood spot
CHSI: Costing of Health System in India
USD: United States dollars
INR: Indian rupee
PSA: Probabilistic sensitivity analysis
CI: Confidence Interval
ANC: Antenatal care
IEC: Information, Education and Communication
HTAIn: Health Technology Assessment in India
ASHA: Accredited Social Health Activist
